# Mechanisms of the anti-aging and prolongevity effects of caloric restriction: evidence from studies of genetically modified animals

**DOI:** 10.18632/aging.101557

**Published:** 2018-09-16

**Authors:** Shunsuke Hoshino, Masaki Kobayashi, Yoshikazu Higami

**Affiliations:** 1 Laboratory of Molecular Pathology and Metabolic Disease, Faculty of Pharmaceutical Sciences, Tokyo University of Science, Noda, Chiba 278-8510, Japan; 2 Translational Research Center, Research Institute for Science and Technology, Tokyo University of Science, Noda, Chiba 278-8510, Japan

**Keywords:** caloric restriction, aging, growth hormone/insulin-like growth factor 1, mitochondria/redox regulation, remodeling of white adipose tissue

## Abstract

It is widely accepted that caloric restriction (CR) extends lifespan and suppresses various pathophysiological changes. CR suppresses growth hormone/insulin-like growth factor signaling and mechanistic target of rapamycin complex 1 activity, activates sirtuin and enhances mitochondrial redox regulation, but the exact mechanisms are still under debate. In this review, we discuss the mechanisms of CR using evidence from studies of animals that were genetically modified according to recent advances in molecular and genetic technologies, from the viewpoint of the adaptive response hypothesis proposed by Holliday (1989). We then explain the beneficial actions of CR, classified according to whether they operate under feeding or fasting conditions.

## Introduction

In 1935, it was reported that caloric restriction (CR) from immediately after weaning extends lifespan in rats [1]. CR, also known as dietary restriction or energy restriction, has been widely applied to aging research as a simple and highly reproducible dietary manipulation to extend lifespan. The prolongevity action of CR has been observed in several species from yeast and nematodes to mammals. In mammals, it has mostly been studied using rodents, in whom CR suppresses various age-associated pathophysiological changes and extends median and maximum lifespan. However, the beneficial actions of CR disappear in certain strains and/or conditions. A recent review by Ingram and de Cabo describes these limitations in detail [2]. For example, CR-induced prolongevity and anti-tumor actions were not observed in F344xBN F1 rats subjected to CR from 18 months of age, but were observed in C3B6F1 mice subjected to CR from 19 months of age. When CR was initiated at 4 months of age, the prolongevity action was observed in C57BL/6Nnia and B6D2F1/Nnia mice, but not in DBA/2Nnia mice. In A/J, C57BL/6J and B6AF1/J mice, CR from 1–2 months of age extended longevity, but CR from 6 months of age did not. Thus, the extent to which CR exerts beneficial effects depends on factors such as species, strain, and timing of CR onset. In general, however, long-term CR from a young age suppresses age-related pathophysiological changes and extends longevity in various rodents. Importantly, moreover, restrictions of individual nutrients (e.g., glucose, lipid, protein) without energy restriction do not induce such beneficial effects [3,4].

More than 20 years ago, it was discovered that Ames dwarf mice, which have a mutation of the *Prop1* gene, live longer than wild-type mice [5]. This was the first report that a single gene mutation or genetic modification was able to extend longevity in mammals. With the recent advances in molecular and genetic technologies, to our knowledge more than 40 mice and rats with a single gene mutation or genetic modification have now been reported to live longer than wild-type animals. Of these mice and rats, approximately one-third show suppressed growth hormone (GH)/insulin-like growth factor 1 (IGF1) signaling. Since CR also suppresses GH/IGF1 signaling, the beneficial actions of CR may involve suppression of GH/IGF1 signaling. Other molecular mechanisms that have been suggested to regulate the beneficial actions of CR include suppression of mechanistic target of rapamycin complex 1 (mTORC1) activity, activation of autophagy, activation of NAD^+^ metabolism and sirtuin, and enhancement of mitochondrial redox regulation [6,7]. However, these mechanisms are not fully understood. Herein, we discuss the mechanisms of CR using evidence from recent studies of genetically modified animals from the viewpoint of the adaptive response hypothesis proposed by Holliday (1989). We then explain the beneficial actions of CR classified according to whether they operate under feeding or fasting conditions.

## Targets and molecular mechanisms of CR

### GH, IGF1 and forkhead box O (FOXO) signaling

GH positively regulates IGF1 production predominantly in liver through a GH receptor (GHR). IGF1 acts on IGF1 receptor and subsequently phosphorylates Akt, a serine/threonine kinase in the target cells. Then, the phosphorylated form of Akt phosphorylates FOXO transcription factors, promoting nuclear export. Therefore, suppression of GH/IGF1 signaling transcriptionally upregulates the expression of several genes activated by FOXO transcription factors.

Ames dwarf, Snell dwarf and GHR knockout (GHR KO) mice show suppressed GH signaling and have extended lifespans. These dwarf mice have similar phenotypes to CR mice, including suppressed GH/IGF1 signaling, reduced thyroid hormone, plasma insulin and glucose, lower body temperature and reduced adiposity. However, the gene expression profile of liver greatly differs between GHR KO mice and CR mice [8]. We have also reported that part of the gene expression profile in the white adipose tissue (WAT) of CR rats is significantly different from that of lifelong dwarf rats bearing the antisense GH transgene [9].

Bonkowski et al. reported that CR enhances insulin sensitivity and extends longevity in wild-type mice but not in GHR KO mice [10]. Therefore, they suggested that the prolongevity action of CR depends on the suppression of GH/IGF1 signaling. In Ames dwarf mice and dwarf rats bearing the antisense GH transgene, CR further extended lifespan [11,12]. These findings suggest that the anti-aging and prolongevity actions of CR might be regulated in both a GH/IGF1 signal dependent and independent manner.

FOXO transcription factors in mammals have four isoforms, i.e. FOXO1, 3, 4 and 6. In FOXO1 KO mice, CR extended lifespan but there was no CR-associated antitumor effect [13]. Conversely, in FOXO3 KO mice, CR suppressed tumorigenesis but there was no CR-induced prolongevity [14]. These differences might be due to the differential activation pattern in tissues and/or cells of the four isoforms of FOXO transcription factors induced by CR.

Brain and muscle aryl like protein 1 (BMAL1) is a transcription factor involved in circadian rhythm. In BMAL1 KO mice, food intake was increased, body weight was reduced and aging phenotypes were accelerated. In these mice, CR did not lower blood IGF1 or insulin levels or extend lifespan, suggesting that BMAL1 is involved in the beneficial action of CR, and that this beneficial action depends on GH/IGF1 signaling [15].

### mTOR signaling

mTOR kinase, a serine/threonine kinase, was identified as a target molecule of rapamycin, an immunosuppressive drug. It forms two distinct multiprotein complexes known as mTORC1 and mTORC2. mTORC1 is widely known to be activated by amino acids and growth factors (e.g., insulin and IGF1). mTORC1 activation promotes protein synthesis via ribosomal protein S6 kinase 1, fatty acid synthesis via sterol regulatory element-binding protein (SREBP) 1, and adipocyte differentiation via peroxisome proliferator-activated receptor gamma (PPARγ), and suppresses autophagy and lysosomal biosynthesis via transcription factor EB (TFEB). In contrast, the function of mTORC2 is poorly understood, but is thought to involve upregulation of anabolic actions and downregulation of catabolic actions, as for mTORC1 [16].

Mice treated with rapamycin, which negatively regulates mTORC1, for a prolonged period after middle age, had extended lifespans [17]. Consistent with this finding, transgenic mice with overexpression of tuberous sclerosis complex 1 (TSC1), which negatively regulates mTORC1, lived longer than wild-type mice [18]. In addition, ribosomal protein S6 kinase KO mice and mTOR mutant mice also lived longer than wild-type mice [19,20].

To our knowledge, the beneficial action of CR has not yet been examined in mice with defective mTORC1 function. In yeast with genetic inhibition of mTOR, however, CR did not extend lifespan [21]. Autophagy is enhanced by mTORC1 suppression. In nematodes deficient in autophagy-related genes, CR did not extend lifespan [22]. On the basis of these findings, it is likely that a decrease in mTOR activity and an activation of autophagic machinery are associated with the beneficial effects of CR.

### Sirtuin

Silent information regulator 2 (*Sir2*) was discovered as a novel gene involved in transcription silencing in yeast. Thereafter, it was reported to play a key role in life extension by CR [23, 24]. Seven orthologs of sirtuin genes, the sirtuins *Sirt1* to *Sirt7*, have been identified in mammals. SIRT1, 6 and 7 proteins are mainly expressed in the nucleus, SIRT2 in the nucleus and cytoplasm, and SIRT 3, 4 and 5 predominantly in mitochondria. The sirtuins catalyze deacetylation reactions of various proteins including histones in a NAD-dependent manner [25].

Among the seven mammalian sirtuins, it is reported that SIRT1, 3 and 6 are involved in age-related pathophysiologies and lifepan [26]. Transgenic mice in which the SIRT1 protein was selectively overexpressed in the hypothalamic neurons had a longer lifespan than wild-type mice, via the orexin type 2 receptor [27]. Transgenic male mice in which the SIRT6 protein was overexpressed had a longer lifespan than wild-type mice [28]. In aged mice, CR for 6 months upregulated SIRT6 expression and ameliorated renal insufficiency. In addition, while overexpression of SIRT6 suppressed cellular senescence via reduced activity of the inflammation-related transcription factor NF-κB, SIRT6 knockdown accelerated cellular senescence [29]. In SIRT3 KO mice, various age-related pathologies occurred earlier [30]. While CR prevented age-related hearing loss in wild-type mice, this effect was not observed in SIRT3 KO mice [31].

### NF-E2-related factor 2 (NRF2)

NRF2 binds to antioxidant response elements to induce expression of target genes in response to oxidation stress and upregulates the expression of genes involved in antioxidant and detoxification responses. Under physiological conditions, NRF2 binds to Kelch-like ECH-associated protein 1 (Keap1) in cytoplasm, where it degrades in the ubiquitin proteasome system. Under conditions of stress including oxidative stress, after Keap1 is trapped by phosphorylated p62, NRF2 translocates to the nucleus, binds to antioxidant response elements and activates the transcription of antioxidant genes [32].

Since the expression of NRF2 decreases with aging in rodents, it is supposed that reactive oxygen species levels and various cancer risks increase. However, CR suppresses the age-related reduction of antioxidant ability by increasing the expression of genes involved in antioxidation and detoxification. In the nematodes, *Skn-1*, a homolog of NRF2, is indispensable to the prolongevity action of CR. NRF2 KO mice exhibit decreased expression of genes involved in antioxidation and detoxification, with the result that oncogenesis is facilitated. The role of NRF2 in the beneficial actions of CR has been examined using NRF2 KO mice. The results showed that NRF2 was important for the antitumor effect of CR but was not involved in the anti-aging, prolongevity or enhanced insulin sensitivity effects of CR [33].

### Neuropeptide Y (NPY)

In mammals, the neurons in the hypothalamic arcuate nucleus sense energy status from the circulation levels of hormones. CR-associated negative energy balance and the subsequent reduction of WAT mass increases circulating ghrelin and adiponectin levels and decreases blood leptin, insulin and IGF1 levels. These hormonal changes activate NPY neurons in the hypothalamic arcuate nucleus. Most of these neurons synthesize agouti-related protein (Agrp), attenuating the activity of the proopiomelanocortin neurons in the arcuate nucleus. The activity change of primary neurons inhibits the second hypothalamic neurons secreting somatotropin, gonadotropin and thyrotropin releasing hormone, and activates neurons secreting corticotropin releasing hormone. This hypothalamic alteration suppresses GH/IGF1 signaling, thyroid function and reproduction, and activates adrenal glucocorticoid function. Most of these altered neuronal secretion profiles are observed in CR mice and rats [34].

In NPY KO mice, CR did not extend longevity, induce tolerance against oxidation stress in the liver, or alter neuronal secretion profile. However, CR did decrease blood insulin and IGF1 levels, increase blood adiponectin and corticosterone levels, and downregulate the expression of genes involved in beta-oxidation in the liver. Therefore, NPY was suggested to be a key factor involved in the GF/IGF1-independent beneficial actions of CR [35].

### Mutation of mitochondrial DNA (mtDNA)

It is accepted that accumulation of mtDNA mutations is one of the key factors of pathogenesis in age-related diseases. PolgA^D257A/D257A^ mice bear a mutation in mtDNA polymerase gamma, and show earlier development of the age-related accumulation of mtDNA mutations and age-associated phenotypes in various tissues [36]. In PolgA^D257A/D257A^ mice, CR did not extend lifespan, suppress accumulation of mtDNA deletion in skeletal muscle or heart, or improve cardiac function, and it worsened sarcopenia. These findings suggest that the accumulation of mtDNA mutations might inhibit the beneficial actions of CR [37].

## Our novel findings: remodeling of WAT by CR

The visceral obesity associated with diabetes, hyperlipidemia and hypertension, known collectively as “metabolic syndrome”, is a known risk factor for life-threating atherosclerotic diseases including myocardial infarction and cerebral infarction. WAT, originally thought to be only an energy storage tissue, has recently been shown to be an endocrine organ, secreting various bioactive molecules called adipokines. Large adipocytes, which accumulate triglyceride (TG) excessively, have increased secretion of inflammatory adipokines, including tumor necrosis factor-α (TNF-α) and interleukin-6 (IL-6), and decreased secretion of adiponectin, compared with small adipocytes, which accumulate less TG. These adipokine secretion profiles are directly involved in age-related pathologies including insulin resistance, hypertension and atherosclerosis [38]. Moreover, it was recently reported that WAT and adipokines are key players in lifespan regulation. For instance, mice with a fat-specific insulin receptor knockout showed reduced adiposity, increased mitochondrial biogenesis and longer lifespans than wild-type mice [39]. Transgenic mice with adiponectin overexpression in the liver showed greater survival than controls [40]. The transcription factors PPARγ and CCAAT/enhancer-binding proteins α (C/EBPα) and β (C/EBPβ) are involved in adipocyte differentiation. Mice with a C/EBPβ gene knock-in at the C/EBPα locus displayed enhanced mitochondrial biogenesis and longer lifespans [41]. In contrast, PPARγ KO mice had shorter lifespans than controls [42].

It is reported that CR increases the active form of adiponectin in mice of any age. This CR-associated upregulation of adiponectin depends on GH/IGF1 signaling [43,44]. We analyzed the CR-associated change chronologically and obtained the following results. CR upregulated the expression of genes and/or proteins involved in fatty acid (FA) biosynthesis and mitochondrial biogenesis in WAT after the early phase of CR, and the CR-associated change occurred more predominantly in WAT than in other tissues or organs. Thereafter, morphological changes occurred, including miniaturization of adipocytes in WAT and metabolic alterations in liver [45]. To clarify the CR-associated metabolic alterations in WAT, which were regulated in a GH/IGF1 signal independent manner, we then compared the gene expression profile in WAT of wild-type CR rats with that of lifelong transgenic dwarf rats fed *ad libitum* (AL). Our results showed that CR upregulated the expression of genes involved in FA biosynthesis, particularly that of a master transcription factor of fatty acid biosynthesis, the SREBP-1 regulatory genes, in a GH/IGF1 independent manner [9].

Therefore, we then compared the CR effect on various parameters including lifespan between SREBP-1c KO mice and wild-type mice. SREBP-1c KO mice had slightly shorter lifespans than wild-type mice. In wild-type mice, CR extended lifespan, increased the expression of proteins involved in FA biosynthesis and mitochondrial biogenesis, and suppressed oxidative stress. Most these changes were observed predominantly in WAT, and not in other tissues. In contrast, the CR-associated life extension and changes in WAT were not observed in SREBP-1c KO mice. It is reported that peroxisome proliferator-activated receptor gamma coactivator-1α (PGC-1α) is a key regulator of CR-induced mitochondrial biogenesis [46]. We observed that SREBP-1c binds to the promoter of the *Pgc-1α* gene, suggesting that SREBP-1c directly regulates *Pgc-1α* transcription [47]. Moreover, results of the proteome analysis of WAT suggested that CR activated the pyruvate/malate cycle [48]. Indeed, it has been reported that CR activates *de novo* FA biosynthesis in WAT but not in liver [45]. These findings indicate that SREBP-1c KO mice cannot effectively utilize lipid under CR conditions. Thus, WAT may not only function as an energy storage tissue, but may also have the role of converting glucose to a more energy dense fatty acid via SREBP-1c under CR conditions.

## Discussion from the viewpoint of the adaptive response hypothesis of CR

**Figure 1:**
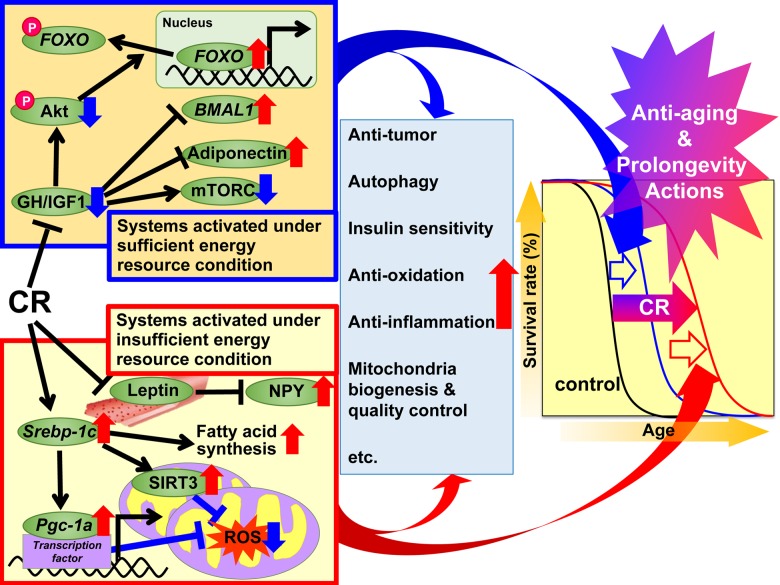
**Proposed mechanisms of the anti-aging and prolongevity actions of caloric restriction (CR) based on the adaptive response hypothesis.** On the basis of the adaptive response hypothesis of CR, we propose that the regulatory mechanisms of CR are classified into two systems, which additively extend lifespan. The first system is activated under sufficient energy resource conditions, when there is grace for free use of energy, and animals grow well, reproduce more, and store excess energy as triglyceride (TG) in white adipose tissue (WAT) for later use, but not to such an extent that they become obese. This system involves growth hormone (GH)/insulin-like growth factor 1 (IGF1), Akt, forkhead box O (FOXO), mechanistic target of rapamycin complex (mTORC), adiponectin and brain and muscle aryl like protein 1 (BMAL1) signaling. In CR animals, these signals act to suppress anabolic reactions. The second system is activated under insufficient energy resource conditions, when there is no grace for free use of energy, and animals suppress growth and reproduction and shift energy use from growth and reproduction to maintenance of biological function, but not to such an extent that they become severely starved. This system involves sterol regulatory element-binding protein 1c (SREBP-1c), sirtuin (SIRT), peroxisome proliferator-activated receptor gamma coactivator-1α (PGC-1α), mitochondrial reactive oxygen species (ROS), leptin and neuropeptide Y (NPY) signaling. In CR animals, these signals act to use energy effectively. Moreover, various signals and/or factors might contribute to CR-associated beneficial actions including anti-oxidative, anti-inflammatory, anti-tumor and other CR actions to a different extent in each tissue or organ, and thereby lead to anti-aging and prolongevity.

In 1989, Holliday explained the anti-aging and prolongevity actions of CR from the evolutionary viewpoint of organisms having evolved adaptive response systems to maximize survival during periods of food shortage [49,50]. On the basis of this evolutionary viewpoint, we divided the beneficial actions of CR into two systems; “systems activated under sufficient energy resource conditions” and “systems activated under insufficient energy resource conditions”. The former is activated under natural environmental conditions that grant animals free use of energy by providing a plentiful food supply. In other words, when there is grace for free use of energy, animals grow well, reproduce more, and store excess energy as TG in WAT for later use, but not to such an extent that they become obese. The latter is activated under natural environmental conditions that do not permit free use of energy because of food shortages. In other words, when there is no grace for free use of energy, animals suppress growth and reproduction and shift energy use from growth and reproduction to maintenance of biological function, but not to such an extent that they become severely starved. Adaptation to natural environmental changes is a top priority for survival in animals. On the basis of the adaptive response hypothesis and the recent findings mentioned above, we propose a suite of mechanisms for the beneficial actions of CR as shown in Figure 1. Since experimental CR conditions can mimic insufficient energy conditions, we hypothesized that CR suppresses “systems activated under sufficient energy conditions” and activates “systems activated under insufficient energy conditions”, and additively induces anti-aging and prolongevity actions. The first set of systems involves GH/IGF1, FOXO, mTORC, adiponectin and BMAL1 signaling, and CR appears to suppress these anabolic reactions. The second set of systems involves SREBP-1c/mitochondria redox, SIRT and NPY signaling, and it is likely that CR activates these reactions to make optimal use of insufficient energy resources. Moreover, various signals and/or factors might contribute to the anti-aging and prolongevity actions of CR to a different extent with anti-oxidative, anti-inflammatory, anti-tumor and other actions in various tissues.

Regarding dietary intervention paradigms, not only CR but also intermittent energy restriction (IER) and time-restricted feeding (TRF) have been applied [2]. IER generally involves fasting every other day or for 2–3 days a week. TRF, which is more popular in obesity research than biogerontology research, generally involves limiting food (high fat diet) access to a few hours per day. The beneficial effects induced by IER or TRF are partly similar to those induced by CR. To our knowledge, however, no studies have used strict research plans including feeding schedules to compare the three dietary interventions. Therefore, comparative studies of CR, IER and TRF might be required in future.

## Prospects

Studies using monkeys suggest that the beneficial actions of CR may occur in humans as well as other mammals [51]. Ongoing CR research focuses on two themes, i.e. elucidation of the molecular mechanisms of CR, and development of CR mimetic medicines. We consider development of novel CR mimetic medicines to be difficult without an understanding of the molecular mechanisms of CR. To develop CR mimetic medicines that are applicable to humans, further studies are therefore required on the molecular mechanisms of CR, particularly in non-human primates. In this report, we propose that the molecular mechanisms of beneficial actions of CR should be classified and discussed according to whether they operate under rich or insufficient energy resource conditions. Future studies of the molecular mechanisms of the beneficial actions of CR should also consider the extent to which the signals/factors involved contribute to the anti-oxidative, anti-inflammatory, anti-tumor and other CR actions in each tissue or organ, and thereby lead to anti-aging and prolongevity. Studies of genetically modified animals focusing on either of the two systems mentioned above showing differences in the extent of CR-induced effects in mice of various backgrounds, and those comparing the beneficial actions of CR with those of IER or TRF, will help to clarify not only the further molecular mechanisms of CR but also those involved in health lifespan.

## References

[1] McCay CM, Crowell MF, Maynard LA. The effect of retarded growth upon the length of life span and upon the ultimate body size. J Nutr. 1935; 1:63–79. 10.1093/jn/10.1.63 2520283

[2] Ingram DK, de Cabo R. Calorie restriction in rodents: caveats to consider. Ageing Res Rev. 2017; 39:15–28. 10.1016/j.arr.2017.05.008 28610949 PMC5565679

[3] Yu BP. Modulation of Aging Processes by Dietary Restriction (1994). CRC Press, Boca Raton, Florida

[4] Weindruch R, Walford RL. The Retardation of Aging and Disease by Dietary Restriction (1988). Charles C Thomas, Springfield, Illinois

[5] Brown-Borg HM, Borg KE, Meliska CJ, Bartke A. Dwarf mice and the ageing process. Nature. 1996; 384:33. 10.1038/384033a0 8900272

[6] López-Otín C, Galluzzi L, Freije JM, Madeo F, Kroemer G. Metabolic control of longevity. Cell. 2016; 166:802–21. 10.1016/j.cell.2016.07.031 27518560

[7] Balasubramanian P, Howell PR, Anderson RM. Aging and caloric restriction research: A biological perspective with translational potential. EBioMedicine. 2017; 21:37–44. 10.1016/j.ebiom.2017.06.015 28648985 PMC5514430

[8] Miller RA, Chang Y, Galecki AT, Al-Regaiey K, Kopchick JJ, Bartke A. Gene expression patterns in calorically restricted mice: partial overlap with long-lived mutant mice. Mol Endocrinol. 2002; 16:2657–66. 10.1210/me.2002-0142 12403853

[9] Chujo Y, Fujii N, Okita N, Konishi T, Narita T, Yamada A, Haruyama Y, Tashiro K, Chiba T, Shimokawa I, Higami Y. Caloric restriction-associated remodeling of rat white adipose tissue: effects on the growth hormone/insulin-like growth factor-1 axis, sterol regulatory element binding protein-1, and macrophage infiltration. Age (Dordr). 2013; 35:1143–56. 10.1007/s11357-012-9439-1 22645024 PMC3705091

[10] Bonkowski MS, Rocha JS, Masternak MM, Al Regaiey KA, Bartke A. Targeted disruption of growth hormone receptor interferes with the beneficial actions of calorie restriction. Proc Natl Acad Sci USA. 2006; 103:7901–05. 10.1073/pnas.0600161103 16682650 PMC1458512

[11] Mattison JA, Wright C, Bronson RT, Roth GS, Ingram DK, Bartke A. Studies of aging in ames dwarf mice: effects of caloric restriction. J Am Aging Assoc. 2000; 23:9–16. 10.1007/s11357-000-0002-0 23604794 PMC3455356

[12] Shimokawa I, Higami Y, Tsuchiya T, Otani H, Komatsu T, Chiba T, Yamaza H. Life span extension by reduction of the growth hormone-insulin-like growth factor-1 axis: relation to caloric restriction. FASEB J. 2003; 17:1108–09. 10.1096/fj.02-0819fje 12692087

[13] Yamaza H, Komatsu T, Wakita S, Kijogi C, Park S, Hayashi H, Chiba T, Mori R, Furuyama T, Mori N, Shimokawa I. FoxO1 is involved in the antineoplastic effect of calorie restriction. Aging Cell. 2010; 9:372–82. 10.1111/j.1474-9726.2010.00563.x 20222901

[14] Shimokawa I, Komatsu T, Hayashi N, Kim SE, Kawata T, Park S, Hayashi H, Yamaza H, Chiba T, Mori R. The life-extending effect of dietary restriction requires Foxo3 in mice. Aging Cell. 2015; 14:707–09. 10.1111/acel.12340 25808402 PMC4531086

[15] Patel SA, Chaudhari A, Gupta R, Velingkaar N, Kondratov RV. Circadian clocks govern calorie restriction-mediated life span extension through BMAL1- and IGF-1-dependent mechanisms. FASEB J. 2016; 30:1634–42. 10.1096/fj.15-282475 26700733 PMC4799504

[16] Pan H, Finkel T. Key proteins and pathways that regulate lifespan. J Biol Chem. 2017; 292:6452–60. 10.1074/jbc.R116.771915 28264931 PMC5399099

[17] Harrison DE, Strong R, Sharp ZD, Nelson JF, Astle CM, Flurkey K, Nadon NL, Wilkinson JE, Frenkel K, Carter CS, Pahor M, Javors MA, Fernandez E, Miller RA. Rapamycin fed late in life extends lifespan in genetically heterogeneous mice. Nature. 2009; 460:392–95. 10.1038/nature08221 19587680 PMC2786175

[18] Zhang HM, Diaz V, Walsh ME, Zhang Y. Moderate lifelong overexpression of tuberous sclerosis complex 1 (TSC1) improves health and survival in mice. Sci Rep. 2017; 7:834. 10.1038/s41598-017-00970-7 28400571 PMC5429778

[19] Selman C, Tullet JM, Wieser D, Irvine E, Lingard SJ, Choudhury AI, Claret M, Al-Qassab H, Carmignac D, Ramadani F, Woods A, Robinson IC, Schuster E, et al. Ribosomal protein S6 kinase 1 signaling regulates mammalian life span. Science. 2009; 326:140–44. 10.1126/science.1177221 19797661 PMC4954603

[20] Wu JJ, Liu J, Chen EB, Wang JJ, Cao L, Narayan N, Fergusson MM, Rovira II, Allen M, Springer DA, Lago CU, Zhang S, DuBois W, et al. Increased mammalian lifespan and a segmental and tissue-specific slowing of aging after genetic reduction of mTOR expression. Cell Reports. 2013; 4:913–20. 10.1016/j.celrep.2013.07.030 23994476 PMC3784301

[21] Kaeberlein M, Powers RW 3rd, Steffen KK, Westman EA, Hu D, Dang N, Kerr EO, Kirkland KT, Fields S, Kennedy BK. Regulation of yeast replicative life span by TOR and Sch9 in response to nutrients. Science. 2005; 310:1193–96. 10.1126/science.1115535 16293764

[22] Jia K, Levine B. Autophagy is required for dietary restriction-mediated life span extension in C. elegans. Autophagy. 2007; 3:597–99. 10.4161/auto.4989 17912023

[23] Kaeberlein M, McVey M, Guarente L. The SIR2/3/4 complex and SIR2 alone promote longevity in Saccharomyces cerevisiae by two different mechanisms. Genes Dev. 1999; 13:2570–80. 10.1101/gad.13.19.2570 10521401 PMC317077

[24] Lin SJ, Defossez PA, Guarente L. Requirement of NAD and SIR2 for life-span extension by calorie restriction in Saccharomyces cerevisiae. Science. 2000; 289:2126–28. 10.1126/science.289.5487.2126 11000115

[25] Houtkooper RH, Pirinen E, Auwerx J. Sirtuins as regulators of metabolism and healthspan. Nat Rev Mol Cell Biol. 2012; 13:225–38. 10.1038/nrm3293 22395773 PMC4872805

[26] Giblin W, Skinner ME, Lombard DB. Sirtuins: guardians of mammalian healthspan. Trends Genet. 2014; 30:271–86. 10.1016/j.tig.2014.04.007 24877878 PMC4077918

[27] Satoh A, Brace CS, Rensing N, Cliften P, Wozniak DF, Herzog ED, Yamada KA, Imai S. Sirt1 extends life span and delays aging in mice through the regulation of Nk2 homeobox 1 in the DMH and LH. Cell Metab. 2013; 18:416–30. 10.1016/j.cmet.2013.07.013 24011076 PMC3794712

[28] Kanfi Y, Naiman S, Amir G, Peshti V, Zinman G, Nahum L, Bar-Joseph Z, Cohen HY. The sirtuin SIRT6 regulates lifespan in male mice. Nature. 2012; 483:218–21. 10.1038/nature10815 22367546

[29] Zhang N, Li Z, Mu W, Li L, Liang Y, Lu M, Wang Z, Qiu Y, Wang Z. Calorie restriction-induced SIRT6 activation delays aging by suppressing NF-κB signaling. Cell Cycle. 2016; 15:1009–18. 10.1080/15384101.2016.1152427 26940461 PMC4889297

[30] McDonnell E, Peterson BS, Bomze HM, Hirschey MD. SIRT3 regulates progression and development of diseases of aging. Trends Endocrinol Metab. 2015; 26:486–92. 10.1016/j.tem.2015.06.001 26138757 PMC4558250

[31] Someya S, Yu W, Hallows WC, Xu J, Vann JM, Leeuwenburgh C, Tanokura M, Denu JM, Prolla TA. Sirt3 mediates reduction of oxidative damage and prevention of age-related hearing loss under caloric restriction. Cell. 2010; 143:802–12. 10.1016/j.cell.2010.10.002 21094524 PMC3018849

[32] Komatsu M, Kurokawa H, Waguri S, Taguchi K, Kobayashi A, Ichimura Y, Sou YS, Ueno I, Sakamoto A, Tong KI, Kim M, Nishito Y, Iemura S, et al. The selective autophagy substrate p62 activates the stress responsive transcription factor Nrf2 through inactivation of Keap1. Nat Cell Biol. 2010; 12:213–23. 10.1038/ncb2021 20173742

[33] Pearson KJ, Lewis KN, Price NL, Chang JW, Perez E, Cascajo MV, Tamashiro KL, Poosala S, Csiszar A, Ungvari Z, Kensler TW, Yamamoto M, Egan JM, et al. Nrf2 mediates cancer protection but not prolongevity induced by caloric restriction. Proc Natl Acad Sci USA. 2008; 105:2325–30. 10.1073/pnas.0712162105 18287083 PMC2268135

[34] Komatsu T, Chiba T, Yamaza H, To K, Toyama H, Higami Y, Shimokawa I. Effect of leptin on hypothalamic gene expression in calorie-restricted rats. J Gerontol A Biol Sci Med Sci. 2006; 61:890–98. 10.1093/gerona/61.9.890 16960019

[35] Chiba T, Tamashiro Y, Park D, Kusudo T, Fujie R, Komatsu T, Kim SE, Park S, Hayashi H, Mori R, Yamashita H, Chung HY, Shimokawa I. A key role for neuropeptide Y in lifespan extension and cancer suppression via dietary restriction. Sci Rep. 2014; 4:4517. 10.1038/srep04517 24682105 PMC3970128

[36] Kujoth GC, Hiona A, Pugh TD, Someya S, Panzer K, Wohlgemuth SE, Hofer T, Seo AY, Sullivan R, Jobling WA, Morrow JD, Van Remmen H, Sedivy JM, et al. Mitochondrial DNA mutations, oxidative stress, and apoptosis in mammalian aging. Science. 2005; 309:481–84. 10.1126/science.1112125 16020738

[37] Someya S, Kujoth GC, Kim MJ, Hacker TA, Vermulst M, Weindruch R, Prolla TA. Effects of calorie restriction on the lifespan and healthspan of POLG mitochondrial mutator mice. PLoS One. 2017; 12:e0171159. 10.1371/journal.pone.0171159 28158260 PMC5291490

[38] Ouchi N, Parker JL, Lugus JJ, Walsh K. Adipokines in inflammation and metabolic disease. Nat Rev Immunol. 2011; 11:85–97. 10.1038/nri2921 21252989 PMC3518031

[39] Otabe S, Yuan X, Fukutani T, Wada N, Hashinaga T, Nakayama H, Hirota N, Kojima M, Yamada K. Overexpression of human adiponectin in transgenic mice results in suppression of fat accumulation and prevention of premature death by high-calorie diet. Am J Physiol Endocrinol Metab. 2007; 293:E210–18. 10.1152/ajpendo.00645.2006 17389708

[40] Blüher M, Kahn BB, Kahn CR. Extended longevity in mice lacking the insulin receptor in adipose tissue. Science. 2003; 299:572–74. 10.1126/science.1078223 12543978

[41] Chiu CH, Lin WD, Huang SY, Lee YH. Effect of a C/EBP gene replacement on mitochondrial biogenesis in fat cells. Genes Dev. 2004; 18:1970–75. 10.1101/gad.1213104 15289464 PMC514177

[42] Argmann C, Dobrin R, Heikkinen S, Auburtin A, Pouilly L, Cock TA, Koutnikova H, Zhu J, Schadt EE, Auwerx J. Ppargamma2 is a key driver of longevity in the mouse. PLoS Genet. 2009; 5:e1000752. 10.1371/journal.pgen.1000752 19997628 PMC2780700

[43] Miller KN, Burhans MS, Clark JP, Howell PR, Polewski MA, DeMuth TM, Eliceiri KW, Lindstrom MJ, Ntambi JM, Anderson RM. Aging and caloric restriction impact adipose tissue, adiponectin, and circulating lipids. Aging Cell. 2017; 16:497–507. 10.1111/acel.12575 28156058 PMC5418198

[44] Gesing A, Al-Regaiey KA, Bartke A, Masternak MM. Growth hormone abolishes beneficial effects of calorie restriction in long-lived Ames dwarf mice. Exp Gerontol. 2014; 58:219–29. 10.1016/j.exger.2014.08.010 25152388 PMC4252511

[45] Okita N, Tsuchiya T, Fukushima M, Itakura K, Yuguchi K, Narita T, Hashizume Y, Sudo Y, Chiba T, Shimokawa I, Higami Y. Chronological analysis of caloric restriction-induced alteration of fatty acid biosynthesis in white adipose tissue of rats. Exp Gerontol. 2015; 63:59–66. 10.1016/j.exger.2015.01.043 25616173

[46] Anderson RM, Barger JL, Edwards MG, Braun KH, O’Connor CE, Prolla TA, Weindruch R. Dynamic regulation of PGC-1alpha localization and turnover implicates mitochondrial adaptation in calorie restriction and the stress response. Aging Cell. 2008; 7:101–11. 10.1111/j.1474-9726.2007.00357.x 18031569 PMC2253697

[47] Fujii N, Narita T, Okita N, Kobayashi M, Furuta Y, Chujo Y, Sakai M, Yamada A, Takeda K, Konishi T, Sudo Y, Shimokawa I, Higami Y. Sterol regulatory element-binding protein-1c orchestrates metabolic remodeling of white adipose tissue by caloric restriction. Aging Cell. 2017; 16:508–17. 10.1111/acel.12576 28256090 PMC5418191

[48] Okita N, Hayashida Y, Kojima Y, Fukushima M, Yuguchi K, Mikami K, Yamauchi A, Watanabe K, Noguchi M, Nakamura M, Toda T, Higami Y. Differential responses of white adipose tissue and brown adipose tissue to caloric restriction in rats. Mech Ageing Dev. 2012; 133:255–66. 10.1016/j.mad.2012.02.003 22414572

[49] Holliday R. Food, reproduction and longevity: is the extended lifespan of calorie-restricted animals an evolutionary adaptation? BioEssays. 1989; 10:125–27. 10.1002/bies.950100408 2730632

[50] Masoro EJ, Austad SN. The evolution of the antiaging action of dietary restriction: a hypothesis. J Gerontol A Biol Sci Med Sci. 1996; 51:B387–91. 10.1093/gerona/51A.6.B387 8914486

[51] Mattison JA, Colman RJ, Beasley TM, Allison DB, Kemnitz JW, Roth GS, Ingram DK, Weindruch R, de Cabo R, Anderson RM. Caloric restriction improves health and survival of rhesus monkeys. Nat Commun. 2017; 8:14063. 10.1038/ncomms14063 28094793 PMC5247583

